# Entomological determinants of insecticide-treated bed net effectiveness in Western Myanmar

**DOI:** 10.1186/1475-2875-12-364

**Published:** 2013-10-11

**Authors:** Frank M Smithuis, Moe Kyaw Kyaw, U Ohn Phe, Ingrid van der Broek, Nina Katterman, Colin Rogers, Patrick Almeida, Piet A Kager, Kasia Stepniewska, Yoel Lubell, Julie A Simpson, Nicholas J White

**Affiliations:** 1 Medical Action Myanmar, Kokkine Swimming Club Lane 32A1, Yangon, Myanmar; 2 Médecins sans Frontières - Holland, Thanlin Road 62A, Yangon, Myanmar; 3 Centre for Infectious Diseases, Tropical Medicine & AIDS, Academic Medical Center, Amsterdam, The Netherlands; 4 Mahidol-Oxford Tropical Medicine Research Unit (MORU), Mahidol University, Faculty of Tropical Medicine, 3rd Floor, 60th Anniversary Chalermprakiat Building, 420/6 Rajvithi Rd., Ratchathewi District, Bangkok 10400, Thailand; 5 Centre for Molecular, Environmental, Genetic & Analytic Epidemiology, Melbourne School of Population and Global Health, The University of Melbourne, Melbourne, Australia; 6 Centre for Tropical Medicine, CCVTM, Churchill Hospital, Oxford, UK

## Abstract

**Background:**

In a large cluster randomized control trial of insecticide-treated bed nets (ITN) in Western Myanmar the malaria protective effect of ITN was found to be highly variable and, in aggregate, the effect was not statistically significant. A coincident entomological investigation measured malaria vector abundance and biting behaviour and the human population sleeping habits, factors relevant to ITN effectiveness.

**Methods:**

Entomological surveys were carried out using different catching methods to identify potential malaria vector species and characterise their biting habits. The salivary glands were dissected from all female anophelines caught to identify sporozoites by microscopy.

**Findings:**

Between 1995 and 2000 a total of 4,824 female anopheline mosquitoes were caught with various catching methods. A total of 916 person nights yielded 3,009 female anopheline mosquitoes between 6 pm and 6 am. Except for *Anopheles annularis*, which showed no apparent preference (51% outdoor biting), all major species showed a strong preference for outdoor biting; *Anopheles epiroticus* (79%), *Anopheles subpictus* (72%), *Anopheles maculatus* (92%), *Anopheles aconitus* (85%) and *Anopheles vagus* (72%). Most human biting occurred in the early evening with the peak biting time between 6 pm and 7 pm (35%). Overall 51% (1447/2837) of all bites recorded were between 6 pm and 8 pm. A large proportion of children were not sleeping under an ITN during peak biting times. Only one *An. annularis* mosquito (0.02%) had malaria sporozoites identified in the salivary glands.

**Conclusions:**

Peak vector biting occurred early in the evening and mainly occurred outdoors. The limited efficacy of ITN in this area of Western Myanmar may be explained by the biting behaviour of the prevalent *Anopheles* mosquito vectors in this area.

## Background

Declining malaria transmission in much of Southeast Asia has led to a transition in policy objectives from one of malaria control to complete elimination [1]. This ‘endgame’ is likely to require more precision in the targeting of interventions. Insecticide-impregnated bed nets (ITN) have proved very effective in highly endemic areas and have contributed substantially to lower childhood malaria morbidity and mortality. Whether ITN are equally beneficial in the context of low, unstable transmission characteristic of much of Southeast Asia, and in moving towards elimination is not well established. The effectiveness of ITN in preventing malaria is dependent on vector abundance, biting behaviour, and the population sleeping habits [2]. In an accompanying report we describe a cluster randomised trial carried out in Western Myanmar to assess the effectiveness of ITN in reducing malaria incidence and improving clinical outcomes in children. ITN provided at best only modest benefit, and overall the differences between ITN and control villages were not statistically significant. In this report we describe the findings of entomological and population sleeping behaviour surveys carried out alongside the large ITN effectiveness study in Western Myanmar that may explain the low malaria protective efficacy observed.

## Methods

The study comparing ITN effectiveness is described in detail in the accompanying paper. In brief, a cluster-randomized controlled trial was conducted in Rakhine State to assess the efficacy of ITN in preventing malaria and anaemia in children and their secondary effects on nutrition and development. The data were aggregated for each village to obtain cluster-level infection rates. In total, 8,175 children under 10 years of age were followed up for 10 months, which included the main malaria transmission period. The incidence and prevalence of *Plasmodium falciparum* and *Plasmodium vivax* infections, and the biting behaviour of *Anopheles* mosquitoes in the area were studied concurrently.

### Entomological surveys

In Rakhine State, 21 species of *Anopheles* have been reported. Among these, *Anopheles dirus* and *Anopheles minimus* were identified as the primary vector species complexes, as in many other parts of the country, while *Anopheles annularis, Anopheles subpictus, Anopheles epiroticus* (previously called *Anopheles sundaicus*), *Anopheles aconitus, Anopheles hyrcanus, Anopheles culicifacies, Anopheles maculatus*, *Anopheles varuna, Anopheles jeyporensis, Anopheles sinensis* and *Anopheles philippinensis* have all been identified as secondary vectors [3-5]. In the nearby Chittagong Hill Tracts in Bangladesh, *Anopheles umbrosus, Anopheles barbirostris, Anopheles nigerrimus*, *Anopheles nivipes, Anopheles kochi*, and *Anopheles vagus* have also been identified as vectors [6].

### Mosquito-trapping methods

Different catching methods were used to identify potential malaria anopheline vector species and determine their biting habits; the degree of anthropophily/zoophily, times of biting and resting habits, i.e. endophily/exophily.

#### Human biting catches (HBC)

Insect collectors were seated inside houses, where people slept and outside in the housing compounds. At each location, one pair of collectors did catches from 6 pm to 12 pm and another pair from 12 pm to 6 am. For the 3rd survey period, the catching period was extended to, start at 5 pm and finish at 7 am, because of the large number of HBC found in the early evening between 6 pm and 7 pm during the previous survey periods. The insect collectors were rotated for inside and outside collections, early and late shifts. At all times, at least one person inside and one person outside the houses was selected from volunteers from the local village population. For outside catches during the rainy season, a simple shelter of plastic sheeting was constructed. The teams were monitored by a supervisor. The collectors caught the mosquitoes landing on their legs (exposed to above the knee) in glass tubes. Hourly catches were labelled and kept separately. The mosquito collectors were offered immediate access to diagnosis and treatment but none reported ill during the survey period.

#### Exit traps

Before sunset, exit traps were attached to the windows of randomly selected houses while all alternative exit-routes were closed by black cotton sheeting, in order to catch the mosquitoes that left the house during the night. Shortly after sunrise the trap was removed and mosquitoes were collected.

#### Knockdown spray

Knockdown sprays collected the indoor-resting population of mosquitoes. Shortly after sunrise, mosquitoes were collected by indoor insecticide spraying (Shell Tox; Fenithrothion 0.15% and Tetramethrin 0.3%). The floors of the rooms were covered with sheets and insecticide was sprayed inside the house and outside the eaves of the roof, with the doors and windows closed. After 10 minutes, the dead mosquitoes were collected from the sheets.

#### Animal biting traps

Cows were placed under non-impregnated bed nets, leaving eaves below of about 10 cm, permitting mosquitoes to enter. Mosquitoes resting on the netting were collected during the night and in the morning.

#### Vector identification

**Figure 1:**
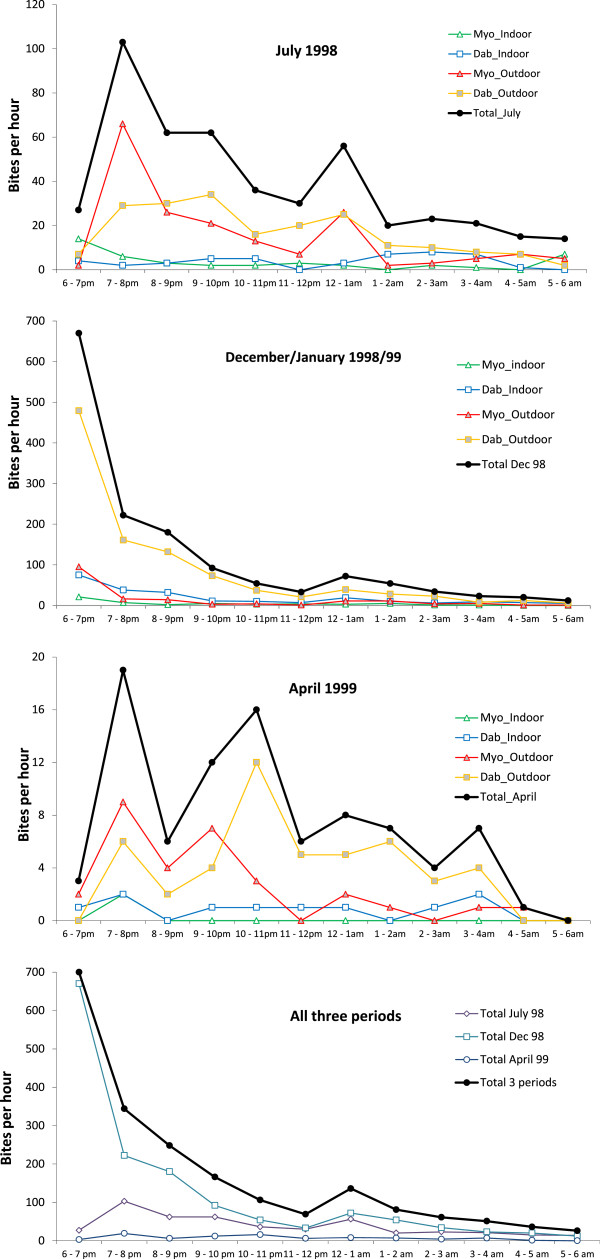
**Biting times of potential vector Anopheles mosquitoes during the different seasons.** Top three panels show summer and winter of 1998 and spring 1999, and bottom panel shows all three catching periods combined. Dab: Dabhine, Myo: Myothugyi.

Species were identified morphologically using published keys and descriptions [3,4,7-9]. From all female *Anopheles,* the salivary glands were dissected to identify the presence of sporozoites by microscopy. Figure 1 in the accompanying paper [10] provides the time frame of entomological surveys carried out between 1995 and 2000 in the study areas.

### Surveys

#### First entomological survey period; November 1995 - April 1996

The first surveys were carried out in November 1995 in Dabhine and in April 1996 in Myothugyi. In Dabhine HBC were performed for 12 person nights. During three nights, one team of four collectors worked simultaneously, two indoors and two outdoors. In addition 33 knockdown spray catches, 11 exit trappings, and three nights of trapping on cattle were performed. In three village tracts of Myothugyi (April 1996), HBC were performed for 40 person nights, during 10 nights with 4 collectors. In addition, 80 knockdown spray-catches (40 houses, two times), and 80 exit-trappings (10 nights, eight traps per night) were carried out.

#### Second entomological survey period; July 1998 - April 1999 (during the ITN trial)

During July 1998 and April 1999 HBC were done during three different seasons: (1) in July; the rainy season, (2) in December-January (15 December – 13 January); the cool season, which is dry and cool at night and (3) in April; the hot season, which is dry and hot [10]. During each season HBC were performed for 192 person nights in total. In both areas where the ITN study was carried out, four villages were selected and matched in pairs with similar environmental conditions. For each pair, one village was selected randomly to receive insecticide-treated bed nets (ITN villages), while the other village acted as control (NN villages). In each village two teams of four persons, two indoors and two outdoors, collected mosquitoes during six nights. One team worked in the evening and the second team worked in the morning.

#### Third entomological survey period; December 1999 - January 2000 (after the ITN trial)

After completion of the ITN study the control villages (NN), had now also received ITNs. Each village pair was now compared with a new, third village, in the same neighbourhood, which did not receive ITNs (New villages). HBC were performed for 288 person nights (22 December – 18 January). Collections were done simultaneously in each of the paired villages during six nights per catching period. HBC were performed in two shifts, from 5–12 pm and from 12–7 am.

#### ITN use and sleeping behaviour related to biting risk

To investigate the effect of incomplete or incorrect usage of ITN, all ITN recipients were interviewed during the cross sectional surveys to assess sleeping times, ITN usage, and washing habits.

### Statistical analysis

The human biting rates were calculated as the number of *Anopheles* biting per person per night (6 pm – 6 am). The human biting rates were calculated over the 2nd survey period only, as catches over this period represented the three seasons, and were therefore a good representation of the year. The EIR was calculated as the product of the human biting rate and the sporozoite rate. Regression was used to investigate the relationship between the malaria incidence and the logarithm of number of vector mosquitoes caught. Correlations between numbers of mosquitoes and malaria incidence were calculated separately for NN and ITN villages using the Spearman rank correlation coefficient. Tests of statistical significance between biting rates in ITN and NN villages could not be performed because the data should be aggregated for each village to obtain cluster-level biting rates and the number of villages was too small to do a cluster analysis.

## Results

### Entomological surveys

**Figure 2:**
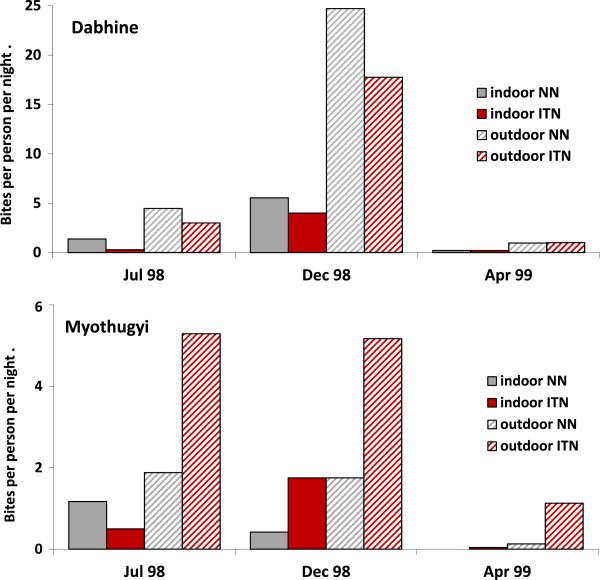
**Numbers of Anopheles mosquitoes caught per person per night in Dabhine and Myothugyi during three seasons.** Upper panel.

In total, 4,824 female anopheline mosquitoes were caught with various catching methods between 1995 and 2000 (Figure 2) (Additional file 1, Additional file 2 and Additional file 3). From 16 November to 1 December 1995, 1382 female *Anopheles* were caught with four different catching methods in the Dabhine area; 94 female *Anopheles* were caught with HBC (32 unfed, 58 blood-fed and four gravid). *Anopheles epiroticus* (six unfed, 33 blood-fed and three gravid) and *An. aconitus* (13 unfed, 20 blood-fed and one gravid) were the predominant species. With the knock-down spray, the main species collected were *An. vagus* (47 blood-fed and 51 gravid), *An. annularis* (51, all blood-fed) and *An. subpictus* (20 blood-fed and 28 gravid). The exit traps sampled small numbers of *An. subpictus* (eight blood-fed and nine gravid), *An. epiroticus* (10; all blood-fed) and *An. annularis* (nine; all blood fed). The cattle biting yielded a high numbers of mosquitoes, in particular of *An. subpictus* (498), *An. barbirostris* (163) and *An. hyrcanus* (152) and to a lesser extent *An. epiroticus* (76) and *An. vagus* (75). All were either blood-fed or gravid when collected.

From 31 March to 12 April 1996, the same catching methods were used in the area around Myothugyi, except for the cattle-biting trap. A total of 78 female *Anopheles* were caught with HBC (45 un-fed, 14 blood fed and 19 gravid). *Anopheles subpictus* was most common (17 unfed, six blood-fed and five gravid). With the knock-down spray, 408 Anopheles were collected (69 unfed, 215 blood-fed and 124 gravid). The main species were *An. subpictus* (59 unfed, 139 blood-fed and 77 gravid) and *An. vagus* (eight unfed, 49 blood-fed and 47 gravid). The exit traps caught 61 Anopheles (41 unfed, five blood-fed and 15 gravid). The main species was *An. subpictus* (32 unfed, two blood-fed and 10 gravid).

During catches in the second and third survey periods, from July 1998 to January 2000, 2895 female anopheline mosquitoes of 16 Anopheles species were caught with HBC. The most commonly caught were *An. epiroticus* (1,427), *An. subpictus* (622), *An. annularis* (342), *An. aconitus* (149), *An. maculatus* (147) and *An. vagus* (116), respectively. These six species combined represented 97% (2,803/2,895) of all female anopheline mosquitoes caught during this period.

More female *Anopheles* were caught in Dabhine (2,177) than in Myothugyi (718). There was considerable variance of species composition between locations and seasons (Additional file 1 and Additional file 2). *Anopheles epiroticus* was the predominant species in both locations and was found mainly during the December/January catches, the peak malaria season. *Anopheles subpictus* and *An. annularis* were found mostly in Dabhine, while *An. maculatus* was caught mainly in Myothugyi. *Anopheles maculatus* and *An. vagus* were predominantly caught in July. *Anopheles dirus* and *An. minimus* were only found in Myothugyi area. One *An. minimus* mosquito was found in a knock down spray catch in 1995 and six *An. dirus* were caught on HBC in July 1998 in Thitawywa, a village in the foothills.

### Human biting rates

A total of 916 person nights yielded 3,009 female anopheline mosquitoes between 6 pm and 6 am. During the first survey period (1995/1996), 94 female *Anopheles* were caught with HBC in Dabhine, during 12 person nights in November 1995 (biting rate; 7.8 bites/pp/night). Twenty-one were caught indoor (indoor biting rate; 3.5 bites/pp/night) and 73 were caught outdoor (outdoor biting rate; 12.2 bites/pp/night). In Myothugyi, a total of 78 female *Anopheles* were caught with HBC during 40 person nights in April 1996 (biting rate; 2.0 bites/pp/night). Eleven mosquitoes were caught indoors (indoor biting rate; 0.6 bites/pp/night) and 67 were caught outdoors (outdoor biting rate 3.6; bites/pp/night).

During the second survey period (July 1998-April 1999) 1985 *Anopheles* were caught with HBC over 576 person nights. The seasonal variation of HBC is considerable (Figure 2), but, if it is assumed that the average of the three HBC episodes (representing the three seasons) is representative for the year, then the average human biting rate is 3.45 bites/pp/night*.*

The largest numbers of mosquitoes (1,466) were caught in December/January 1998/99, during the dry-cool season, (biting rate; 7.6 bites/pp/night). In Dabhine, 1,248 mosquitoes were caught in 96 person-nights (biting rate; 13.0 bites/pp/night) and, in Myothugyi, 218 mosquitoes were caught (biting rate; 2.3 bites/pp/night). During the rainy season (July 1998) 431 mosquitoes were caught in 192 person-nights (biting rate; 2.2 bites/pp/night). In Dabhine 219 *Anopheles* were caught (biting rate 2.3 bites/pp/night) and in Myothugyi 212 *Anopheles* were caught (biting rate 2.2 bites/pp/night). In April 1999, during the dry-hot season, 88 mosquitoes were caught in 192 person-nights (biting rate 0.5 bites/pp/night). In Dabhine, 57 *Anopheles* were caught (biting rate; 0.6 bites/pp/night), and, in Myothugyi, 31 *Anopheles* were caught (biting rate; 0.3 bites/pp/night).

In the 3rd survey period (December 1999/January 2000), 852 mosquitoes were caught in 288 person-nights (biting rate; 3.0 bites/pp/night). In Dabhine, 605 mosquitoes (biting rate; 4.2 bites/pp/night) and, in Myothugyi, 247 mosquitoes were caught (biting rate; 1.7 bites/pp/night) (6 pm – 6 am). Additional file 3 provides an overview of all human bite catches between 1995 and 2000 in the study areas.

### Endo and exophagy

To define the level of endophagy and exophagy the proportion of HBC indoors and outdoors was compared (Additional file 4) from 664 (23%) indoor catches and 2173 (77%) outdoor catches. Except for *An. annularis*, which showed no apparent preference (51% outdoor biting), all major species showed a strong preference for outdoor biting; *An. epiroticus* (79%), *An. subpictus* (72%), *An. maculatus* (92%), *An. aconitus* (85%) and *An. vagus* (72%) (Table 1).

**Table 1:** **Biting times and location of female *Anopheline mosquitoes *** **during the 2nd and 3rd survey periods (1998–2000)**

**Species**	**Indoor biting (%)**	**Outdoor biting (%)**	**Before 8 pm (%)**	**After 8 pm (%)**
*An. aconitus*	21 (15%)	122 (85%)	52 (36%)	91 (64%)
*An. annularis*	165 (49%)	171 (51%)	73 (21%)	263 (79%)
*An. maculatus*	12 (8%)	135 (92%)	46 (31%)	101 (69%)
*An. subpictus*	119 (20%)	481 (80%)	312 (52%)	288 (48%)
*An. epiroticus*	294 (21%)	1111 (79%)	869 (61%)	536 (39%)
*An. vagus*	31 (27%)	84 (73%)	57 (34%)	58 (66%)
Other *Anopheles*	22 (24%)	69 (76%)	38 (39%)	53 (61%)
Total	664 (23%)	2173 (77%)	1447 (51%)	1390 (49%)

*Note* excluding catches during 5-6 pm and 6-7 am in December 1999.

### Biting times

**Figure 3:**
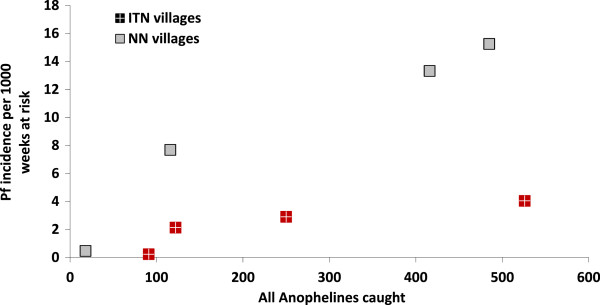
The relationship between the incidence of falciparum malaria and potential vector Anopheles mosquitoes caught on human bite catches.

Between July 1998 and January 2000, all HBC (2,837, not including catches before 6 pm and after 6 am) were recorded hourly, between 6 pm and 6 am, (Figure 1). Most biting occurred in the early evening with the peak biting time between 6 pm and 7 pm (992 HBC, 35%). Overall 51% (1,447/2,837) of all HBC recorded were made in the two hours between 6 pm. and 8 pm. Early biting was particularly common among *An. epiroticus* (61% before 8 pm) and least likely among *An. annularis* (21% before 8 pm). The pattern of early biting was most marked during the high transmission cool season, during which 57% (1,315/2,318) of HBCs were before 8 pm. In Dec/Jan 1998/99 and in Dec/Jan 1999/2000 61% (892/1,466) and 50% (423/852) respectively of HBC were before 8 pm. During July 1998 and April 1999 the mosquitoes were also active at later hours during the evening and only 26% (111/431) bit before 8 pm in July 1998 and 24% (21/88) bit before 8 pm in April 1999 (Figure 3). The proportion of outdoor mosquito catches before 8 pm (53%; 1151/2173) was significantly higher than the proportion of indoor biting before 8 pm (45%; 296/664), p = 0.0001. In Dabhine 50% (1060/2129) of all HBC had been caught before 20.00 hrs; 53% (855/1628) of outdoor catches and 41% (205/501) of indoor catches were before 8 pm, p < 0.0001. Whereas in Myothugyi 55% (387/708) of all HBC had been caught before 20.00 hrs; 54% (296/545) of outdoor catches and 56% (91/163) of indoor catches, p = 0.773, (Table 1).

Because of the very early biting pattern observed in December/January 1998/99, the last catching period (Dec/Jan 1999/2000) was extended starting at 5 pm and finishing at 7 am. During this period 910 mosquitoes were caught: 852 between 6 pm and 6 am, 37 (4%) *Anopheles* mosquitoes were caught between 5 and 6 pm (14 *An. epiroticus*, 15 *An. subpictus*, four *An. aconitus*, three *An. annularis* and one *An. vagus*), and 20 (2%) were caught between 6 and 7 am (eight *An. epiroticus*, seven *An. subpictus*, three *An. annularis*, two *An. aconitus* and one *An. jamesi*). However, as this was not done in the previous periods, these HBC were not included in the biting rates and calculations of biting times.

### Entomological inoculation rate

Of 4,824 female *Anopheles* mosquitoes caught between 1995 and 2000, only one *An. annularis* mosquito (0.02%) had malaria sporozoites in the salivary glands. The single infected mosquito was captured in Dabhine in December 1998, during outdoor HBC between 6 pm and 7 pm in a village where ITN had been distributed. During all HBC, 349 *An. annularis* were collected and dissected, thus the estimated sporozoite prevalence of *An. annularis* found on HBC was 0.29% (95% CI; 0 to 0.85%). The other species caught were *An. epiroticus* (1,469), *An. subpictus* (654), *An. aconitus* (192), *An. maculatus* (147) and *An. vagus* (132), respectively. Thus the upper 95% CI for sporozoite prevalences for other potential vectors were *An. epiroticus* (0.20%), *An. subpictus* (0.46%), *An. aconitus* (1.56%), *An. maculatus* (2.04%) and *An. vagus* (2.27%), respectively. To calculate the entomological inoculation rate (EIR), HBC from villages that did not have ITN should be used ideally, as ITN could have influenced the mosquito population and the infection rate. Nevertheless assuming that both the number of mosquitoes and the sporozoite rates were not influenced by ITNs the numbers of HBC in villages with and without ITNs were combined The overall sporozoite prevalence for biting anophelines was 1/3,067 or 0.0326% (95% CI 0 – 0.0965). With an estimated biting rate of 3.45 bites per person per night the annual EIR is estimated as 0.0326% × 3.45 × 365 = 0.41 infective bites per person per year (95% CI = 0 – 1.22).

### Mosquito behaviour and densities in ITN and non-ITN villages

To compare biting frequencies HBC (1985) were evaluated during the second survey period, July 98 (rainy season, intermediate transmission), December/January 1998/99 (cool season, peak transmission) and April 99 (hot and dry season, low transmission), because in this period the HBC was performed equally in four ITN and four NN villages. In NN villages, 1,022 HBC (indoor + outdoor) were made in 288 person-nights (biting rate 3.5 pp/night) while in the ITN villages 963 HBC were made during 288 nights (biting rate 3.3 pp/night). In Dabhine region, fewer *Anopheles* were caught in ITN villages (630) than in villages without ITN (894) while, in Myothugyi, this trend was the opposite; more *Anopheles* were caught in villages with ITN (333) than in villages without ITN (128) (Additional file 4 and Additional file 5). Indoor biting was 163/963 (17%) in ITN villages and 209/1,022 (20%) in NN villages. The early biting pattern was also similar in villages with ITN (496/963 or 52% before 8 pm) and villages without ITN (528/1022 or 52% before 8 pm). In Dabhine region, the biting rate (indoor and outdoor combined) increased markedly from July 1998 (2.28) to December 1998 (13.0). This occurred both in ITN villages (from 1.6 to 10.9) and NN villages (from 2.9 to 15.1). This trend was similar for indoor and outdoor human biting.

In Myothugyi region, the biting rates (pp/night) were similar in July 1998 (2.2) and December 1998 (2.3). In ITN villages the biting rate was 2.9 in July and 3.5 in December. In NN villages these rates were respectively 1.5 and 1.1. Indoor biting in ITN villages increased from July (0.4) to December (2.8) while indoor biting in NN villages also increased from July (1.3) to December (2.98). In April HBC in all villages in both regions decreased sharply compared to the December/January catches.

### Correlation between *Anopheles* abundance and falciparum malaria

The correlation coefficient (r_s_) between the incidence of falciparum malaria and the number of female *Anopheles* caught on human bite catches was 0.7, (p = 0.07); in ITN villages (r = 0.9, p = 0.13) and in control villages (r = 1, p = 0.04) (Figure 3). Regarding specific species, the strongest correlation between falciparum malaria incidence and *Anopheles* species was found for *An. annularis* and *An. epiroticus* (and to a lesser extent for *An. subpictus* and *An. vagus*). The relationship between the incidence of falciparum malaria and the number of female *Anopheles* caught was investigated by multiple linear regression modelling (Additional file 6). Slopes in the regression model were significantly different between ITN and NN villages (p < 0.001, likelihood ratio test) : 9.98 in the control villages and 4.40 in the bed net villages thus for the same number of mosquitoes caught, as there were twice as many malaria cases observed in the control villages compared with the bed net villages.

### ITN use and sleeping behaviour related to biting risk

In order to investigate the effect of incomplete or incorrect usage of ITN we interviewed all ITN recipients, during the cross sectional surveys, regarding ITN usage and ITN washing habits. After the first study period from May to September, 14 out of 3,948 persons (0.35%) said that they had washed their nets, and 24 out of 3,964 persons (0.61%) said that they did not always use the net. During the 2nd period from September to February 1536 out of 3,861 persons (39.8%) said that they had washed their ITN and 158/3,894 (4%) said that they did not always use it. After excluding 208 people for whom data were incomplete, 3,857 persons remained for analysis. 1,646 were defined as poor ITN users, i.e. they mentioned that they had washed their ITN or had not always used them. Among the so-called “poor bed net users” 130 children (7.9%) had one or more *P. falciparum* infection while 195 children (8.8%) among the “good bed net users” had a *P. falciparum* infection. For *P. vivax,* these figures were 188 (11.4%) among poor ITN users versus 197 (8.9%) among good users.

In order to assess the potential protective effect of ITN, behaviour was observed before sleeping and the time spent in bed of people in the studied villages. A total of 503 person-night scores were obtained. Six person night scores were excluded from the analysis, where the person involved was away collecting mosquitoes, and 18 person night scores because sleeping data were incomplete or because they were not sleeping at their home. In villages where ITN were distributed, 286/341 (84%) of the persons slept under their ITN while in the control villages 10/138 (7%) of people used their own – usually non-impregnated bed nets. To calculate the risk of being bitten according to age, a further 52 scores were excluded because the behaviour of the person before bed time was not recorded. For all age groups, 49% (209/427) stayed inside the house and 51% (218/427) stayed outside, before they went to bed. Behaviour before sleeping (indoors or outdoors) and sleeping time was clearly related to age. The majority of children under five years of age (33 out of 53 observed evenings; 62%) remained inside the house before sleeping. Of children between five and nine years, 45 out of 65 evenings observed (69%) were spent outside and for the 10–14 age group (43 evenings) and persons 15 years and older (110 evenings) respectively 66% and 45% of the evenings were spent outside.

**Figure 4:**
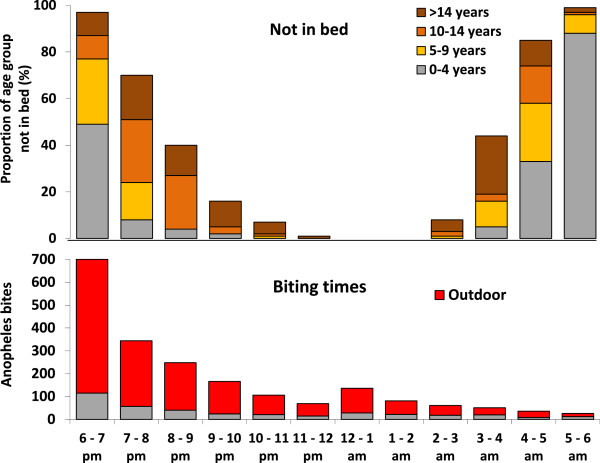
**Comparison of sleeping behaviour of different age-groups and biting times of Anopheles mosquitoes.** The biting time data are based on 2024 human bite catches in 3 seasons.

Children <5 years and the majority of children aged 5 to 9 years went to sleep between 6 pm and 8 pm, whereas the older children and adults stayed up later. In the morning, the adults typically awoke between 4 and 5 am, while the children woke up later. During the night, 39% (167/424) of the people left the house once and 3% (12/424) twice. The different sleeping behaviour by age is particularly relevant considering the biting times of *Anopheles* mosquitoes (Figure 4).

The risk of being bitten for an average person was calculated using the reported behaviour pattern before sleeping (inside or outside the house), the sleeping times, and the entomological data (biting frequencies per hour, indoor versus outdoor biting). For this calculation it was assumed that all persons in the ITN-villages were protected from mosquitoes by ITN during their sleep, while the non-ITN-villagers were exposed to indoor-biting mosquitoes. Table 2 gives the total risk in mosquito bites per night for each age group. Young children (0–4) in both areas had least ‘exposure’ to mosquito-bites and thus least risk of being bitten because they stayed mainly indoors, whilst most biting occurred outdoors (Figure 4). Therefore, the biting-risk of 0–4 years old children in NN-villages was about 61% in Dabhine and 91% in Myothugyi compared to an average person (all age-groups combined). In ITN villages, assuming that ITN prevent biting completely during sleep, infants also obtained a higher level of protection from ITN than the other age groups, because they slept longer. Compared to an average person in a village without ITN, the risk of mosquito-bites per infant was 19% in Dabhine and 89% in Myothugyi.

**Table 2:** Calculated risk of mosquito-bites per person per night per age-category, compared to an average person in a village without ITN in two study areas

**Dabhine**	**NN**			**ITN**	
	**N**	**Bites/night**	**Ratio**	**Bites/night**	**Ratio**
Age 0 – 4 yr	19	2.66	0.61	0.83	0.19
Age 5 – 9 yr	24	4.02	0.93	1.66	0.38
Age 10 – 14 yr	29	4.36	1.01	2.07	0.48
Age >14	113	4.68	1.08	2.45	0.57
All age groups	185	4.34	1.00	2.12	0.49
**Myothugyi**	**NN**			**ITN**	
	**N**	**Bites/night**	**Ratio**	**Bites/night**	**Ratio**
Age 0 – 4 yr	34	0.56	0.91	0.55	0.89
Age 5 – 9 yr	41	0.59	0.94	0.69	1.12
Age 10 – 14 yr	36	0.66	1.06	1.02	1.64
Age >14	131	0.64	1.03	0.99	1.59
All age groups	242	0.62	1.00	0.88	1.42

## Discussion

The relatively poor efficacy of ITN in this study is most likely explained by the early evening biting and exophilic behaviour of the malaria vectors. Very early evening biting was particularly prominent during the dry cool season, the main transmission period in this area, while evening biting in the wet season and dry-hot season was not confined to a narrow crepuscular time window. Temperature influences the flying and feeding activities of mosquitoes. When temperatures fall below 20°C, flying and feeding activity decreases significantly. In the cool dry season in December/January in this study area, the temperature before 5.00 pm is around 25°C. After sunset, at approximately 5.30 pm, temperatures fall quickly to levels below 20°C after 7.00 pm. These cool temperatures are clearly not an impediment to sporozoite development (sporogony) as the malaria incidence peaks during this season. Similar observations have been made during field studies in Punjab Province, Pakistan (approx. 32°N), where Reisen & Aslamkhan [10] found that the peak of biting was markedly crepuscular during periods of low ambient temperature (i.e. a peak at 6.00 to 7.00 pm in November to February) but this shifted with increasing ambient temperatures to around 10.00 to 11.00 pm in May to July. In areas where malaria transmission season peaks during the cool season, early biting can be expected, and ITN protective efficacy may be reduced.

Biting between 5 and 6 pm was considerable and was often higher than at other times between 6 pm and 6 am. This suggests that routine human biting catches should start before 6 pm in areas where early-evening biting is common, particularly for cool season evaluations of ITN preventive efficacy. Outdoor biting was more common (77%) than indoor biting. Outdoor biting was significantly earlier than indoor biting, probably because of the colder temperatures outdoors than indoors.

There was a strong correlation between the number of Anopheles mosquitoes caught in villages and the incidence of falciparum malaria. *Anopheles epiroticus* (n = 1469) was the most commonly caught *Anopheles* species in human bait catches (48%) in both areas and, as in other coastal areas of South-East Asia, is likely to be an important malaria vector in this area [5,11-13]. The Rakhine state is composed of a network of inland seawater rivers and streams and tidal creeks along these rivers. Saline prawn ponds are being cultivated nearby the villages. This forms an ideal habitat for *An. epiroticus,* which likes to breed in brackish water. *Anopheles epiroticus* can also develop in fresh water, and it was found also in villages on the other side of the foothills, where no brackish water is found. In December 1998, when malaria incidence was very high, *An. epiroticus* was responsible for 72% of the human bite catches. The correlation between the prevalence of *An. epiroticus* and falciparum malaria incidence in the study villages was strong. However, this species has not yet been found infected with *Plasmodium* in Myanmar and in this study no sporozoites were found in 1,567 *An. epiroticus* caught. Suspecting anopheline species as vectors based on high human biting rates can be misleading [14] and this survey cannot confirm *An. epiroticus* as the main vector. As *An. epiroticus* bit exceptionally early mainly outdoors it is unlikely to be seriously affected by ITN use. *Anopheles subpictus,* which appears to be an important vector in Sri Lanka [15], was also common, mainly in the Dabhine area. It was collected throughout the year but also thrived during the peak season (December). *Anopheles subpictus* strongly preferred outdoor (79%) and early (61% <8 pm) biting. It was also caught in large numbers on cattle and it is known to be strongly zoophilic. The correlation between *An. subpictus* and falciparum malaria incidence was weak, and it is unlikely to be an important vector in this region. *An. annularis* (n = 349) was the third most frequently caught Anopheles species on HBC (11%) and the only species found with sporozoites (albeit only once). The species of malaria parasite was not identified. *Anopheles annularis* was almost exclusively found in Dabhine region. The correlation between the prevalence of *An. annularis* and falciparum malaria incidence in the study villages was very strong (r = 0.8, p = 0.008) and it is, therefore, likely that *An. annularis* does play a role in malaria transmission in the Dabhine region. However, the human bite catches of *An. annularis* were rather low during the peak malaria season in December 1998, which makes a role as the major vector less likely. In December 1999, *An. annularis* was surprisingly frequent in the new villages (n = 193). Of these, 192 were caught in one ‘new’ village linked to pair 1. *Anopheles annularis* was the only vector that bit equally indoors as well as outdoors and it did not have the early biting pattern of the other species. It was also found in the knock-down spray and exit trap catches and it is therefore more likely to be affected by ITN. During human bite catches less *An. annularis* were collected in ITN villages compared to control villages, while, with the exception of *An. vagus,* this trend was not seen for the other vectors. But the numbers were small, variation in mosquito populations among clusters was large, and it would be premature to draw firm conclusions from these data. *Anopheles aconitus* (n = 192) was found most villages in both regions, but the numbers were also small. *Anopheles aconitus* strongly favoured outdoor biting (85%). The correlation with falciparum malaria incidence was weak. *Anopheles maculatus*, (n = 147), a well-established malaria vector elsewhere in the region [6,14,16,17], was almost entirely found in Myothugyi (146) in two villages on the foothills, during July 1998 catches (142). It strongly favours outdoor biting (92%). It showed a very poor correlation with falciparum malaria, but that could be because it was caught only in three villages. Early evening biting was less prominent for this species. *Anopheles vagus* (132) was also mainly caught in July 1998 (101). Early biting was less prominent. The correlation between the prevalence of *An. vagus* and falciparum malaria incidence in the study villages was quite strong. *Anopheles dirus* and *An. minimus,* which were identified previously as the main vector species in Rakhine State, and are the main vectors in many other areas of Southeast Asia, were almost absent in the study areas [3-6,11,16-22]. In Myothugyi, the lower transmission area, only one *An. minimus* mosquito was found in a knock down spray catch in 1995 and six *An. dirus* were caught on HBC in July 1998, in Thitawywa, a village in the foothills. In Dabhine, a higher transmission area, *An. dirus* and *An. minimus* were not found at all. These are forest and forest-fringe vectors, which do not appear to play an important role in malaria transmission in this area. *Anopheles hyrcanus (HBC; 5), An. culicifacies* (HBC; 0), *An. varuna* (HBC; 7), which were previously identified as subsidiary vectors were also detected in small numbers, and *An. hyrcanus* (152) was linked to cattle.

Of all 4,866 anopheles mosquitoes caught, only one was identified with sporozoites, limiting assessment of the relative importance of malaria vectors. Compared to the use of PCR, microscopy is a relatively insensitive method to detect sporozoites and the prevalence of mosquito salivary gland sporozoite carriage estimated in this study is probably an underestimation [23]. Therefore, the EIR is also likely to be an underestimation, but it is still much lower than might be expected from the pattern of malaria clinical epidemiology. The discrepancy presumably results from the previous lack of availability of effective anti-malarial drugs. In a low transmission setting where malaria is generally untreated, then infection prevalence and consequent splenomegaly rates can be similar to that seen in much higher transmission areas where effective drugs are available.

Little can be said about the effects of ITN on the mosquito population. In Dabhine, fewer mosquitoes were caught with human bite catches both indoor and outdoor in ITN villages than in the control villages, but in Myothugyi the opposite trend was found. The differences between ITN villages and control villages were small and there was a large variance of species composition per location and season. Coincidently the village pairs involved with the entomological studies were also the pairs with the largest differences in falciparum malaria incidence in favour of ITN. The overall rate difference between ITN and NN villages was 3.7 falciparum malaria infections per 1,000 person weeks, while in the four village pairs where the entomology survey was done, the rate difference was 6.9 infections per 1,000 person weeks. The numbers of mosquitoes caught in ITN and NN villages cannot be compared because randomization was per cluster, the inter-cluster variability was very large, and the number of clusters was too low for adequate comparison.

Biting was less frequent in the winter of 1999/2000 than in the winter of 1998/99 both in the ITN and NN villages which by then had also received ITN. This might have resulted from ITN use, but in other malaria programme areas in Rakhine State where ITN were not distributed, a similar pattern of malaria cases was observed; 8,233 malaria patients were treated in December 1998 and January 1999 versus 5,519 malaria patients in December 1999 and January 2000. In the new villages, where ITN were not distributed, human biting catches were also lower than in the same period of the year before. Thus variance between years cannot be excluded as the main factor for the lower biting frequency in the winter of 1999/2000. To find out if ITN decrease biting rates in this area, mosquito catching should be continued for several years.

The effectiveness of ITN in preventing malaria depends on vectors biting when most people are in or near bed. In Africa, where transmission of malaria is generally higher and most malaria vectors bite late at night or early in the morning, ITN have proved very successful, and they have rightly become the cornerstone of malaria control. In Western Myanmar, only *An. annularis* bit throughout the night and bit equally indoors and outdoors, which makes it a reasonable target for vector control by insecticide treated nets. *Anopheles annularis* may be an important malaria vector in Dabhine area, but not in Myothugyi area, which could have contributed to the better performance of ITN in Dabhine. Any mass effects provided were not sufficient to reduce malaria significantly. In East Asia there is enormous complexity in the malaria vectors and their behaviour, although many do bite predominantly outdoors and early in the evening or morning. This diminishes the benefits of ITNs in preventing malaria, and as in this area, makes early diagnosis and effective anti-malarial treatment a more cost-effective intervention.

## Competing interests

The authors declare that they have no competing interests.

## Authors’ contributions

FS, JAS and NJW conceived the idea for the paper. JAS, KS, MKK and FS conducted the analysis. YL conducted the economic evaluation. FS and NJW wrote the paper with input from all other authors. All authors have approved the final version of this paper.

## Supplementary Material

Additional file 1 **Anophelines captured during first survey period.** Female anopheline mosquitoes collected during catches in the 1st survey period (1995–1996), in Dabhine (Dab) and Myothugyi (Myo) using different trapping methods. Click here for file

Additional file 2 **Anophelines captured during second survey period.** Indoor and outdoor human bite catches of female *Anopheles* mosquitoes during the 2nd survey period, comparing villages with and without insecticide-treated bed nets. Click here for file

Additional file 3 **Overview of human bite catches.** An overview of human bite catches performed in the study villages during the reported study periods, in Dabhine and Myothugyi using different trapping methods. Click here for file

Additional file 4 **Effects of ITN on indoor and outdoor biting.** Indoor and outdoor human bite catches of female *Anopheles* mosquitoes during the 2nd survey period, comparing villages with and without insecticide-treated bed nets. Click here for file

Additional file 5 **Effects of ITN on anopheline biting assessed by HBC.** Human bite catches of female *Anopheline mosquitoes* according to season and village type during the 2nd and 3rd survey periods (1998 – 2000). Click here for file

Additional file 6 **Relationship between falciparum malaria incidence and anophelines captured.** Linear regression model for the incidence of falciparum malaria (number of episodes per 1000 weeks) in relation to the number of *Anopheles* caught. Click here for file

## References

[B1] Cui L Yan G Sattabongkot J Chen B Cao Y Fan Q Parker D Sirichaisinthop J Su XZ Yang H Yang Z Wang B Zhou G Challenges and prospects for malaria elimination in the Greater Mekong Subregion Acta Trop 2012 12 240 245 10.1016/j.actatropica.2011.04.006 21515238 PMC3155744

[B2] Stewart T Marchand RP Factors that affect the success and failure of insecticide-treated net programs for malaria control in SE Asia and the Western Pacific 2003 Geneva: World Health Organization 1 36

[B3] Oo TT The biology and vector competence of the Anopheline mosquitoes in Myanmar with special consideration of Anopheles dirus Doctor of Science Dissertation 2003 Heidelberg: University of Heidelberg http://archiv.ub.uni-heidelberg.de/volltextserver/3305/1/Thesis.pdf

[B4] Oo TT Storch V Becker N Review of the anopheline mosquitoes of Myanmar J Vector Ecol 2004 12 21 40 15266738

[B5] Alam MS Chakma S Khan WA Glass GE Mohon AN Elahi R Norris LC Podder MP Ahmed S Haque R Sack DA Sullivan DJ Jr Norris DE Diversity of anopheline species and their plasmodium infection status in rural Bandarban, Bangladesh Parasit Vectors 2012 12 e10 10.1186/1756-3305-5-10 PMC3419674 22839212

[B6] Puri M Synoptic table for the identification of the anopheline mosquitoes of India Health Bulletin No-10 malaria bureau, no-2 1957 5 Delhi: Government of India, Manager of Publications

[B7] Delphin F Rao V Guide for the identification of the full-grown larvae and imagines of the anophelines of Burma 1957 Rangoon: Union of Burma

[B8] Das BP Rajagopal R Akiyama J Zoology; pictorial key to the species of Indian anopheline mosquitoes J Pure Applied Zool 1990 12 131 162

[B9] Smithuis FM Kyaw MK Phe UO van der Broek I Katterman N Rogers C Almeida P Kager PA Stepniewska K Lubell Y Simpson JA White NJ The effect of insecticide-treated bed nets on the incidence and prevalence of malaria in children in an area of unstable seasonal transmission in western Myanmar Malaria Journal 2013 12 363 10.1186/1475-2875-12-363 24119916 PMC3854704

[B10] Reisen WK Aslamkhan M Biting rhythms of some Pakistan mosquitoes (Diptera: Culicidae) Bull Ent Res 1978 12 313 330 10.1017/S0007485300007392

[B11] Van Bortel W Chinh VD Berkvens D Speybroeck N Trung HD Coosemans M Impact of insecticide-treated nets on wild pyrethroid resistant *Anopheles epiroticus* population from southern Vietnam tested in experimental huts Malar J 2009 12 248 10.1186/1475-2875-8-248 19874581 PMC2781025

[B12] Trung HD Van Bortel W Sochantha T Keokenchanh K Quang NT Cong LD Coosemans M Malaria transmission and major malaria vectors in different geographical areas of Southeast Asia Trop Med Int Health 2004 12 230 237 10.1046/j.1365-3156.2003.01179.x 15040560

[B13] Verhaeghen K Van Bortel W Trung HD Sochantha T Coosemans M Absence of knockdown resistance suggests metabolic resistance in the main malaria vectors of the Mekong region Malar J 2009 12 e84 10.1186/1475-2875-8-84 PMC2679768 19400943

[B14] Trung HD Van Bortel W Sochantha T Keokenchanh K Briët OJT Coosemans M Behavioural heterogeneity of *Anopheles* species in ecologically different localities in Southeast Asia: a challenge for vector control Trop Med Int Health 2005 12 251 262 10.1111/j.1365-3156.2004.01378.x 15730510

[B15] Amerasinghe PH Amerasinghe FP Wirtz RA Indrajith NG Somapala W Malaria transmission by *Anopheles subpictus* (Diptera: Culicidae) in a new irrigation project in Sri Lanka J Med Entomol 1992 12 577 581 1495065 10.1093/jmedent/29.4.577

[B16] Vector Borne Disease Control (VBDC) Annual reports 1982–2000 1982–2000 Myanmar: Unpublished reports of Department of Health

[B17] Yu G Yan G Zhang N Zhong D Wang Y He Z Yan Z Fu W Yang F Chen B The Anopheles community and the role of *Anopheles minimus* on malaria transmission on the China-Myanmar border Parasit Vectors 2013 12 e264 10.1186/1756-3305-6-264 PMC3856509 24034528

[B18] Ahmed S Galagan S Scobie H Khyang J Prue CS Khan WA Ram M Alam MS Haq MZ Akter J Glass G Norris DE Nyunt MM Shields T Sullivan D Sack DA Malaria hotspots drive hypoendemic transmission in the Chittagong Hill Districts of Bangladesh PLoS One 2013 12 e69713 10.1371/journal.pone.0069713 23936345 PMC3735545

[B19] Thang ND Erhart A Speybroeck N Xa NX Thanh NN Ky PV Hung Le X Thuan Le K Coosemans M D’Alessandro U Long-lasting insecticidal hammocks for controlling forest malaria: a community-based trial in a rural area of central Vietnam PLoS One 2009 12 e7369 10.1371/journal.pone.0007369 19809502 PMC2752990

[B20] Sochantha T Van Bortel W Savonnaroth S Marcotty T Speybroeck N Coosemans M Personal protection by long-lasting insecticidal hammocks against the bites of forest malaria vectors Trop Med Int Health 2010 12 336 341 10.1111/j.1365-3156.2009.02457.x 20070632

[B21] Morel CM Thang ND Erhart A Xa NX Peeters Grietens K Xuan Hung L Thuan Le K Van Ky P Hung NM Coosemans M D’Alessandro U Mills A Cost-effectiveness of long-lasting insecticide-treated hammocks in preventing malaria in South-central Vietnam PLoS One 2013 12 e58205 10.1371/journal.pone.0058205 23536790 PMC3594234

[B22] Rattanarithikul R Konishi E Linthicum KJ Detection of *Plasmodium vivax* and *Plasmodium falciparum* circumsporozoite antigen in anopheline mosquitoes collected in southern Thailand Am J Trop Med Hyg 1999 12 114 121 10.4269/ajtmh.1996.54.114 8619432

[B23] Snounou G Pinheiro L Goncalves A Fonseca L Dias F Brown KN Do Rosario VE The importance of sensitive detection of malaria parasites in the human and insect host in epidemiological studies, as shown by the analysis of field samples from Guinea Bissau Trans R Soc Trop Med Hyg 1993 12 649 653 10.1016/0035-9203(93)90274-T 8296364

