# Supplementary material for: Entomological determinants of insecticide-treated bed net effectiveness in Western Myanmar
Source: Malar J. 2013 Oct 11;12:364. doi: 10.1186/1475-2875-12-364 (PMC4015723; doi:10.1186/1475-2875-12-364)
Supplement: Additional file 1 — Anophelines captured during first survey period. Female anopheline mosquitoes collected during catches in the 1st survey period (1995–1996), in Dabhine (Dab) and Myothugyi (Myo) using different trapping methods. [file 1475-2875-12-364-S1.docx]

Additional file 1. Female anopheline mosquitoes collections during catches in the 1^st^ survey period (1995-1996), in Dabhine (Dab) and Myothugyi (Myo) using different trapping methods.

|  | Human biting rate (bites/pp/pn)  Indoor | | Human biting rate (bites/pp/pn)  Outdoor | | Exit Trap | | Knock Down  Spray | | Cattle Trap | Total mosquitoes caught |
| --- | --- | --- | --- | --- | --- | --- | --- | --- | --- | --- |
|  | Dab | Myo | Dab | Myo | Dab | Myo | Dab | Myo | Dab |  |
| An. aconitus | 1.83 | 0 | 3.83 | 0.45 | 0 | 0.04 | 0.06 | 0.21 | 6.33 | 84 |
| *An. annularis* | 0.67 | 0.05 | 0.17 | 0.05 | 0.82 | 0 | 1.55 | 0 | 2.33 | 74 |
| *An. barbirostris* | 0 | 0 | 0.17 | 0 | 0.09 | 0.03 | 0 | 0.07 | 54.33 | 173 |
| *An. hyrcanus* | 0 | 0 | 0.50 | 0 | 0.09 | 0 | 0 | 0 | 50.67 | 156 |
| *An. jamesii* | 0 | 0.25 | 0 | 0.55 | 0 | 0 | 0 | 0 | 3.33 | 26 |
| *An. jeyporiensis* | 0 | 0 | 0 | 0.10 | 0 | 0.03 | 0 | 0.02 | 2.33 | 13 |
| *An. minimus* | 0 | 0 | 0 | 0 | 0 | 0 | 0 | 0.01 | 0 | 1 |
| *An. philippinensis* | 0 | 0.10 | 0 | 0.05 | 0 | 0 | 0.12 | 0 | 0.33 | 8 |
| *An. subpictus* | 0.17 | 0 | 0.50 | 1.40 | 1.55 | 0.59 | 1.45 | 3.35 | 166.00 | 914 |
| *An. epiroticus* | 0.67 | 0 | 6.33 | 0 | 0.91 | 0.01 | 0.30 | 0.01 | 25.33 | 140 |
| *An. tessellatus* | 0.17 | 0 | 0.67 | 0.05 | 0 | 0 | 0 | 0.01 | 1.00 | 10 |
| *An. Vagus* | 0 | 0.15 | 0 | 0.65 | 0 | 0.09 | 2.97 | 1.27 | 25.00 | 300 |
| *An. varuna* | 0 | 0 | 0 | 0.05 | 0.36 | 0.03 | 0 | 0.01 | 7.33 | 30 |
| Total *Anopheles* | 3.50 | 0.55 | 12.17 | 3.35 | 3.82 | 0.81 | 6.45 | 4.98 | 344.33 | 1929 |

Dabhine (November 1995): human biting catches; 12 person nights, knock down spray; 33 collections, exit traps; 11 traps, cattle trap; 3 nights. Myothugyi (April 1996): human biting catches; 40 person nights, exit trap; 75 traps, knock down spray; 82 collections. For exit trap, knock down spray and cattle trap, figures represent the average of the trapping method. Bites per person per night.
