# Supplementary material for: Entomological determinants of insecticide-treated bed net effectiveness in Western Myanmar
Source: Malar J. 2013 Oct 11;12:364. doi: 10.1186/1475-2875-12-364 (PMC4015723; doi:10.1186/1475-2875-12-364)
Supplement: Additional file 2 — Anophelines captured during second survey period. Indoor and outdoor human bite catches of female Anopheles mosquitoes during the 2nd survey period, comparing villages with and without insecticide-treated bed nets. [file 1475-2875-12-364-S2.docx]

**Additional file 2.** Human biting rate (bites/person/night) of female *Anopheles* mosquitoes in Dabhine and Myothugyi during the 2^nd^ and 3^rd^ survey periods (1998-2000).

|  | Dabhine | | | | | | | | Myothugyi | | | | | | | | Total mosquitoes caught |
| --- | --- | --- | --- | --- | --- | --- | --- | --- | --- | --- | --- | --- | --- | --- | --- | --- | --- |
|  | Indoor | | | | Outdoor | | | | Indoor | | | | Outdoor | | | |  |
|  | July 98 | Dec 98 | Apr 99 | Dec 99 | July 98 | Dec 98 | Apr 99 | Dec 99 | July 98 | Dec 98 | Apr 99 | Dec 99 | July 98 | Dec 98 | Apr 99 | Dec 99 |  |
| *An. aconitus* | 0 | 0.02 | 0.04 | 0.08 | 0.06 | 0.29 | 0.35 | 0.72 | 0.10 | 0.15 | 0 | 0.03 | 0.06 | 0.33 | 0.02 | 0.28 | 149 |
| *An. annularis* | 0.60 | 0.38 | 0.06 | 1.64 | 0.98 | 0.40 | 0.10 | 1.42 | 0 | 0 | 0 | 0 | 0.02 | 0 | 0 | 0 | 342 |
| *An. barbirostris* | 0 | 0 | 0 | 0 | 0 | 0 | 0 | 0 | 0 | 0 | 0 | 0 | 0 | 0 | 0 | 0.01 | 1 |
| *An. Dirus* | 0 | 0 | 0 | 0 | 0 | 0 | 0 | 0 | 0.02 | 0 | 0 | 0 | 0.10 | 0 | 0 | 0 | 6 |
| *An. hyrcanus* | 0 | 0 | 0 | 0 | 0 | 0 | 0 | 0.01 | 0 | 0 | 0 | 0 | 0 | 0 | 0 | 0 | 1 |
| *An. jamesii* | 0 | 0 | 0 | 0 | 0 | 0 | 0 | 0.01 | 0 | 0.04 | 0 | 0 | 0 | 0.10 | 0 | 0.06 | 12 |
| *An. jeyporiensis* | 0 | 0 | 0 | 0 | 0 | 0 | 0 | 0 | 0.02 | 0.13 | 0 | 0.13 | 0.06 | 0.19 | 0 | 0.08 | 34 |
| *An. karwari* | 0 | 0 | 0 | 0 | 0 | 0 | 0 | 0 | 0 | 0 | 0 | 0 | 0 | 0.06 | 0 | 0.08 | 9 |
| *An. kochi* | 0 | 0 | 0 | 0 | 0 | 0 | 0 | 0 | 0 | 0 | 0 | 0 | 0.02 | 0.02 | 0 | 0 | 2 |
| *An. maculates* | 0 | 0.02 | 0 | 0 | 0 | 0 | 0 | 0 | 0.19 | 0 | 0 | 0.03 | 2.77 | 0 | 0.02 | 0.01 | 147 |
| *An. philippinensis* | 0 | 0 | 0 | 0.01 | 0 | 0 | 0 | 0.01 | 0 | 0 | 0 | 0.01 | 0.06 | 0.17 | 0 | 0.03 | 16 |
| *An. subpictus* | 0.17 | 0.83 | 0.10 | 1.11 | 1.48 | 5.21 | 0.50 | 1.58 | 0 | 0 | 0 | 0.03 | 0.02 | 0.06 | 0.31 | 0.13 | 622 |
| *An. epiroticus* | 0 | 3.52 | 0 | 0.56 | 0.04 | 15.27 | 0 | 1.86 | 0 | 0.73 | 0.02 | 0.75 | 0.04 | 2.48 | 0.25 | 1.75 | 1427 |
| *An. tessellates* | 0 | 0 | 0 | 0 | 0 | 0.06 | 0 | 0 | 0 | 0 | 0 | 0 | 0.02 | 0 | 0 | 0.01 | 5 |
| *An. Vagus* | 0.06 | 0 | 0 | 0.01 | 1.17 | 0 | 0.02 | 0.03 | 0.50 | 0.02 | 0 | 0.04 | 0.38 | 0 | 0.02 | 0.08 | 116 |
| *An. Varuna* | 0 | 0 | 0 | 0 | 0 | 0 | 0 | 0 | 0 | 0.02 | 0 | 0 | 0.02 | 0.04 | 0 | 0.03 | 6 |
| Total | 0.83 | 4.77 | 0.21 | 3.42 | 3.73 | 21.23 | 0.98 | 5.65 | 0.83 | 1.08 | 0.02 | 1.01 | 3.58 | 3.46 | 0.63 | 2.56 | 2895 |

Numbers of July 1998, December 1998 and April 1999 give the total catches in 4 villages per region (48 person-nights indoor and 48 person-nights outdoor). December/Jan 1999/2000 catches sum catches of 6 villages per region (72 person-nights indoor and 72 person-nights outdoor) and includes biting from 5pm to 7 am.
