# Supplementary material for: Entomological determinants of insecticide-treated bed net effectiveness in Western Myanmar
Source: Malar J. 2013 Oct 11;12:364. doi: 10.1186/1475-2875-12-364 (PMC4015723; doi:10.1186/1475-2875-12-364)
Supplement: Additional file 3 — Overview of human bite catches. An overview of human bite catches performed in the study villages during the reported study periods, in Dabhine and Myothugyi using different trapping methods. [file 1475-2875-12-364-S3.docx]

Additional file 3. An overview of human bite catches performed in the study villages during the reported study periods, in Dabhine and Myothugyi using different trapping methods.

|  | | |  | Dabhine | | Myothugyi | | Total |
| --- | --- | --- | --- | --- | --- | --- | --- | --- |
|  | Villages  (No) | person nights | catching  time | indoor | outdoor | indoor | outdoor |  |
| Nov 95 | 1 | 12 nights | 6pm-6am | 21 | 73 |  |  | 94 |
| Apr 96 | 1 | 40 nights | 6pm-6am |  |  | 11 | 67 | 78 |
|  |  |  |  |  |  |  |  |  |
| July 98 | 4 ITN, 4 NN | 192 nights | 6pm-6am | 40 | 179 | 40 | 172 | 431 |
| Dec 98 | 4 ITN, 4 NN | 192 nights | 6pm-6am | 229 | 1019 | 52 | 166 | 1466 |
| Apr 99 | 4 ITN, 4 NN | 192 nights | 6pm-6am | 10 | 47 | 1 | 30 | 88 |
|  |  |  |  |  |  |  |  |  |
| Dec 99 | 4 ITN, 4 NN and 4 NEW | 288 nights | 6pm-6am | 222 | 383 | 70 | 177 | 852 |
|  |  |  | 5-6pm & 6-7am | (24) | (24) | (3) | (7) | (58) |
| Total catches between 6pm and 6am | | | | 522 | 1701 | 174 | 612 | 3009 |
| (including catches between 5-6pm and 6-7am in Dec 99) | | | | (546) | (1725) | (177) | (619) | (3067) |

ITN: insecticide treated nets; NN: No nets.
