# Supplementary material for: Entomological determinants of insecticide-treated bed net effectiveness in Western Myanmar
Source: Malar J. 2013 Oct 11;12:364. doi: 10.1186/1475-2875-12-364 (PMC4015723; doi:10.1186/1475-2875-12-364)
Supplement: Additional file 4 — Effects of ITN on indoor and outdoor biting. Indoor and outdoor human bite catches of female Anopheles mosquitoes during the 2nd survey period, comparing villages with and without insecticide-treated bed nets. [file 1475-2875-12-364-S4.docx]

**Additional file 4.** Indoor and outdoor human bite catches of female *Anopheles* mosquitoes during the 2^nd^ survey period, comparing villages with and without insecticide treated bednets.

|  | Dabhine (1524) | | | | Myothugyi (461) | | | |
| --- | --- | --- | --- | --- | --- | --- | --- | --- |
|  | ITN | | NN | | ITN | | NN | |
|  | Indoor | Outdoor | Indoor | Outdoor | Indoor | Outdoor | Indoor | Outdoor |
| *An. Aconitus* | 1 | 16 | 2 | 18 | 10 | 14 | 2 | 6 |
| *An. Annularis* | 5 | 16 | 45 | 55 | . | . | . | 1 |
| An. maculates | . | . | 1 | . | 7 | 114 | 2 | 20 |
| *An. subpictus* | 27 | 173 | 26 | 172 | . | 18 | . | 1 |
| *An. epiroticus* | 75 | 297 | 94 | 438 | 29 | 105 | 7 | 28 |
| *An. vagus* | . | 19 | 3 | 38 | . | 3 | 25 | 16 |
| other *An.* species | 0 | 1 | 0 | 2 | 9 | 24 | 2 | 18 |
| All species | 108 | 522 | 171 | 723 | 55 | 278 | 38 | 90 |

ITN: insecticide treated nets; NN: No nets.
