# Supplementary material for: Entomological determinants of insecticide-treated bed net effectiveness in Western Myanmar
Source: Malar J. 2013 Oct 11;12:364. doi: 10.1186/1475-2875-12-364 (PMC4015723; doi:10.1186/1475-2875-12-364)
Supplement: Additional file 5 — Effects of ITN on anopheline biting assessed by HBC. Human bite catches of female Anopheline mosquitoes according to season and village type during the 2nd and 3rd survey periods (1998 – 2000). [file 1475-2875-12-364-S5.docx]

**Additional file 5.** Human bite catches of female *Anopheline mosquitoes* according to season and village type during the 2^nd^ and 3^rd^ survey periods (1998 – 2000).

|  | July 98 | | Dec 98 | | Apr 99 | | Dec 99^a^ | | |
| --- | --- | --- | --- | --- | --- | --- | --- | --- | --- |
|  | ITN | NN | ITN | NN | ITN | NN | ITN | NN ^b^ | New |
| *An. aconitus* | 7 | 4 | 21 | 17 | 13 | 7 | 14 | 16 | 44 |
| *An. annularis* | 12 | 65 | 3 | 34 | 6 | 2 | 0 | 21 | 193 ^c^ |
| *An. maculatous* | 120 | 22 | . | 1 | 1 | . | 1 | 0 | 2 |
| *An. subpictus* | 47 | 33 | 147 | 146 | 24 | 20 | 27 | 55 | 101 |
| *An. epiroticus* | 3 | 1 | 491 | 565 | 12 | 1 | 87 | 77 | 168 |
| *An. vagus* | 21 | 80 | . | 1 | 1 | 1 | 4 | 6 | 1 |
| other species | 8 | 8 | 26 | 14 | 0 | 0 | 10 | 5 | 20 |
| Total | 218 | 213 | 688 | 778 | 57 | 31 | 143 | 180 | 529 |

^a^ Dec 1999 catching before 6pm and after 6 am are excluded. ^b^ The NN received ITN in June 1999. ^c^ 192 *An. annularis* were caught in 1 village

ITN: insecticide treated nets; NN: No nets.
