# Supplementary material for: Entomological determinants of insecticide-treated bed net effectiveness in Western Myanmar
Source: Malar J. 2013 Oct 11;12:364. doi: 10.1186/1475-2875-12-364 (PMC4015723; doi:10.1186/1475-2875-12-364)
Supplement: Additional file 6 — Relationship between falciparum malaria incidence and anophelines captured. Linear regression model for the incidence of falciparum malaria (number of episodes per 1000 weeks) in relation to the number of Anopheles caught. [file 1475-2875-12-364-S6.docx]

**Additional file 6**; Linear regression model for the incidence of falciparum malaria (number of episodes per 1000 weeks) in relation to the number of *Anopheles* caught.

|  | regression coefficient | SE | p-value |
| --- | --- | --- | --- |
| Slope for ITN village | 4.404 | 1.163 | 0.063 |
| Intercept for ITN | -7.765 | 2.686 | 0.102 |
| Slope for NN village | 9.982 | 0.693 | 0.005 |
| Intercept for NN | -12.346 | 1.545 | 0.015 |
| Test for interaction (difference in slopes) ; |  |  | 0.0003 |

SE = standard error, ITN: insecticide treated nets; NN: No nets.
